# Evaluation of lung tumor motion in a large sample: Target‐related and clinical factors influencing tumor motion based on four‐dimensional CT

**DOI:** 10.1002/cam4.4255

**Published:** 2021-09-14

**Authors:** Fengxiang Li, Yanlin Qu, Tingting Zhang, Zhen Cui, Xin Sun, Tao Zhang, Jianbin Li

**Affiliations:** ^1^ Cheeloo College of Medicine Shandong University Jinan China; ^2^ Department of Radiation Oncology Shandong Cancer Hospital and Institute Shandong First Medical University and Shandong Academy of Medical Sciences Jinan China; ^3^ Department of Biostatistics School of Public Health Cheeloo College of Medicine Shandong University Jinan China; ^4^ Institute for Medical Dataology Cheeloo College of Medicine Shandong University Jinan China

**Keywords:** clinical factors, four‐dimensional CT, lung tumor, stereotactic body radiation therapy, target‐related factors, tumor motion

## Abstract

**Background and purpose:**

We aimed to analyze the influence of target‐related and clinical factors on lung tumor motion based on four‐dimensional CT (4DCT), and clarify the motion based on subgroups in lung stereotactic body radiation therapy.

**Materials and methods:**

4DCT image data of 267 tumors from 246 patients were analyzed. The coordinates in the left–right (LR), anterior–posterior (AP), and cranial–caudal (CC) directions of the center of mass (COM) of the gross tumor volumes in 10 phases of 4DCT were measured. The peak‐to‐peak COM displacement in the LR, AP, CC, and 3D directions was calculated. The influence of target‐related and clinical factors on tumor motion was evaluated using multivariate analysis.

**Results:**

The tumor segment location correlated with the tumor motion in each direction. Tumor size was predictive of tumor motion in the 3D (*p* = 0.023) and AP directions (*p* = 0.049). The tumor motion for metastatic tumors was smaller than that for primary tumors in the LR (*p* = 0.019) and AP directions (*p* = 0.008). The CC motion for pulmonary surgery recipients (3.8 ± 4.5 mm) was less than that for patients who had not undergone surgery (5.6 ± 5.4 mm), and no significant clinical factor was observed. BSA and BMI were positively correlated with the motion in the CC (*p* = 0.02) and LR directions (*p* = 0.002).

**Conclusion:**

The tumor segment location was a good predictor of tumor motion. A larger tumor tends to have a smaller motion. Patients with metastatic tumors or those who have undergone pulmonary surgery exhibited smaller and more unpredictable tumor motions, which required individual assessments. Thus, clinical factors can potentially predict tumor motion.

## Graphical abstract

This study evaluated the tumor motion magnitude in different lung lobes and segments in a large sample. The purpose of the study was to systematically analyze the influence of the target‐related and clinical factors on tumor motion in different directions and to clarify the tumor motion magnitude for different subgroups of patients.

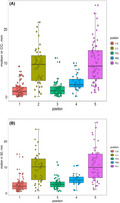

## 1 INTRODUCTION

Stereotactic body radiation therapy (SBRT) has become the standard of care for medically inoperable patients with early stage non‐small cell lung cancer (NSCLC),^1^, ^2^ and has shown significant efficacy in pulmonary oligometastases in patients with lung cancer, whether or not they undergo resection.^3^, ^4^, ^5^, ^6^ High biological dose delivery to patients requires a high conformal dose distribution around the target.^7^

Respiratory‐induced tumor motion is a well‐established cause of inter‐fraction and intra‐fraction geometric uncertainty during radiation delivery.^8^, ^9^ RTOG0813 and RTOG0915 trials recommended a 0.5‐cm margin in the axial plane and a 1.0‐cm margin in the longitudinal plane to accounting for tumor motion based on conventional 3DCT for lung SBRT.^10^, ^11^ The uniform margin may not represent the individual tumor motion, and result in a geographical miss or normal tissue unnecessarily irradiated, which may lead to high risk of radiation‐related side effects.^2^ Therefore, accurately accounting for tumor motion and generating individual internal target volume (ITV) is essential for the success of SBRT.

Four‐dimensional CT (4DCT) is considered a reliable tool to simulate respiration‐induced intrapulmonary motion,^12^, ^13^, ^14^ and individual 4DCT‐based ITVs are widely used in lung SBRT. For a single patient, whether a 4DCT scanning could provide a reliable tumor motion for treatment has been controversial.^15^, ^16^ The tumor motion magnitude could be influenced by target‐related (e.g., size and location) and clinical factors (tumor origin and history of pulmonary surgery).^17^ Understanding the tumor motion magnitude for different patient subgroups and using this information in constructing ITVs is crucial. Moreover, the motion feature could be used to review the reliability of tumor motion for a specific patient measured by multiple manners.^17^

Previous studies concluded that the cranial–caudal (CC) location significantly influences tumor motion.^17^, ^18^, ^19^, ^20^ However, the impact of target size on tumor motion remains controversial.^17^, ^18^, ^19^ Moreover, the influence of clinical factors has not been explicitly elaborated due to relatively small samples. The differences in the tumor motion between pulmonary primary and metastatic tumors, and between patients who previously underwent pulmonary surgery and those who did not remains unclear.

Herein, we evaluated the tumor motion magnitude in different lung lobes and segments in a large sample. This study aimed to systematically analyze the influence of the target‐related and clinical factors on tumor motion in different directions and to clarify the tumor motion magnitude for different subgroups of patients. The information obtained may be valuable in reviewing the reliability of the ITV for individual patients, and providing motion data for generating individual ITVs to patients with limited access to 4DCT scanning.

## 2 MATERIALS AND METHODS

### 2.1 Patient selection and characteristics

This study was a retrospective analysis, approved by the ethics board of Shandong Cancer Hospital and Institute, and the need for participants’ informed consent was waived. In total, 246 of 438 patients who underwent lung SBRT between May 2015 and December 2019 at Shandong Cancer Hospital and Institute were enrolled, 15 of whom had multiple tumors; we included the image data of 267 tumors. Our inclusion criteria were: (1) peripheral lung tumors or metastases; (2) 4DCT images of adequate quality; and (3) CT‐identifiable gross tumor volume (GTV). Our exclusion criteria were: (1) missing 4DCT images; (2) extensive and diffuse tumors; or (3) tumor boundary not easily distinguishable from surrounding pneumonia.

### 2.2 CT simulation and image acquisition

Patients were immobilized using vacuum bags or the Body Pro‐Lok ONE^TM^ system (CIVCO, Coralville, IA) in the supine position with their arms raised above their head. A conventional 3DCT scan of the thoracic region was performed, followed by a 4DCT scan during free breathing on a Brilliance Bores CT simulator (Philips Medical Systems). The 3DCT and 4DCT acquisition protocols were as previously reported.^21^, ^22^ The 4DCT images were sorted into 10 bins according to the breathing signal phase, with 0% corresponding to end‐inhalation and 50% corresponding to end‐exhalation. The CT images were reconstructed using a thickness of 3 mm or 2 mm (tumor diameter ≤1 cm), then transferred to the Eclipse treatment planning system (Varian Medical Systems). Three‐dimensional conformal radiotherapy or intensity‐modulated radiation therapy treatments were planned based on conventional 3DCT for lung SBRT.

### 2.3 Target volume contouring

GTVs were contoured based on each of the 10 phases of 4DCT. All contours were performed by an experienced radiation oncologist using the following contouring protocol: (1) GTVs were delineated using a standard lung gray‐scale window setting in the Aria Eclipse environment (Varian Medical Systems)^23^; (2) the use of the standard mediastinum window was allowed for information purposes to avoid including adjacent vessels and mediastinal or chest wall structures; and (3) blurring in the tumor periphery, representing the “partial volume effect” and “partial projection effect for moving objects,” was included in the GTVs.^24^

### 2.4 Tumor motion

The coordinates in the left–right (LR), anterior–posterior (AP), and cranial–caudal (CC) directions of the center of mass (COM) of the GTVs in 10 4DCT phases were measured. The peak‐to‐peak COM displacement in the three directions was calculated based on the coordinates representing tumor motion. The 3D motion vector (vector) of the COM was calculated as follows:

$$3D\;\text{vector}\;=\;\sqrt{{\text{LR}}^{2}+{\text{AP}}^{2}+{\text{CC}}^{2}}$$

### 2.5 Target‐related and clinical factors

Target‐related factors included the size and location (lobes, segments, abutment relation, and zones). The end‐exhalation of 4DCT‐derived GTV size represented the target size. The lungs are divided into 10 segments according to Gray's Anatomy. The abutment relationship referred to solitary pulmonary tumors, and tumors adjacent to the chest wall, the mediastinum, or the diaphragm. Zoning referred to the interior, intermediate, and lateral third zones of the ipsilateral lung. Clinical factors included patient sex, age, body mass index (BMI), body surface area (BSA), Karnofsky Performance Scale (KPS), smoking history, pathology, pulmonary surgery history, and coexisting pulmonary disease, heart disease, hypertensive disease, or diabetes.

### 2.6 Statistical analyses

The distribution of the tumor motion in the LR, AP, CC, and 3D directions for different lobes was represented using the box plot. Log transform was used to normalize the tumor motion. Multiple linear regression models were used to explore the tumor motion‐related risk factors in the LR, AP, CC, and 3D directions. Akaike information criterion‐based backward stepwise regression was used to identify important variables. While metastatic tumor and surgery were identified as protective factors, stratification analyses were used to explore the different effects of selected variables in patients with metastatic tumors and in surgery recipients. The individuals were divided into the primary and the metastatic tumor groups and characteristics were described. Variables were described using mean (SD), median [IQR], and n (%), as appropriate. The difference in these variables was assessed by a two‐sample *t*‐test, Wilcoxon rank‐sum test, and chi‐squared or Fisher exact test, as appropriate. All analyses were performed using R version 4.0.4. The hypothesis tests were two‐sided, and *p* < 0.05 was considered statistically significant.

## 3 RESULTS

We analyzed the image data of 267 tumors. The mean tumor motion amplitudes were 1.5 ± 1.2, 2.2 ± 1.5, 5.3 ± 5.3, and 6.4 ± 5.2 mm in the LR, AP, CC, and 3D directions, respectively. The median motion amplitudes were 1.2 (0.1–11.4), 1.9 (0.3–11), 3.2 (0.1–27.1), and 4.4 mm (0.6–27.28 mm) in the LR, AP, CC, and 3D directions, respectively. We discovered that 95% of the tumors moved less than 3.7, 5.1, 16.4, and 16.7 mm in the LR, AP, CC, and 3D directions, respectively.

**FIGURE 1:**
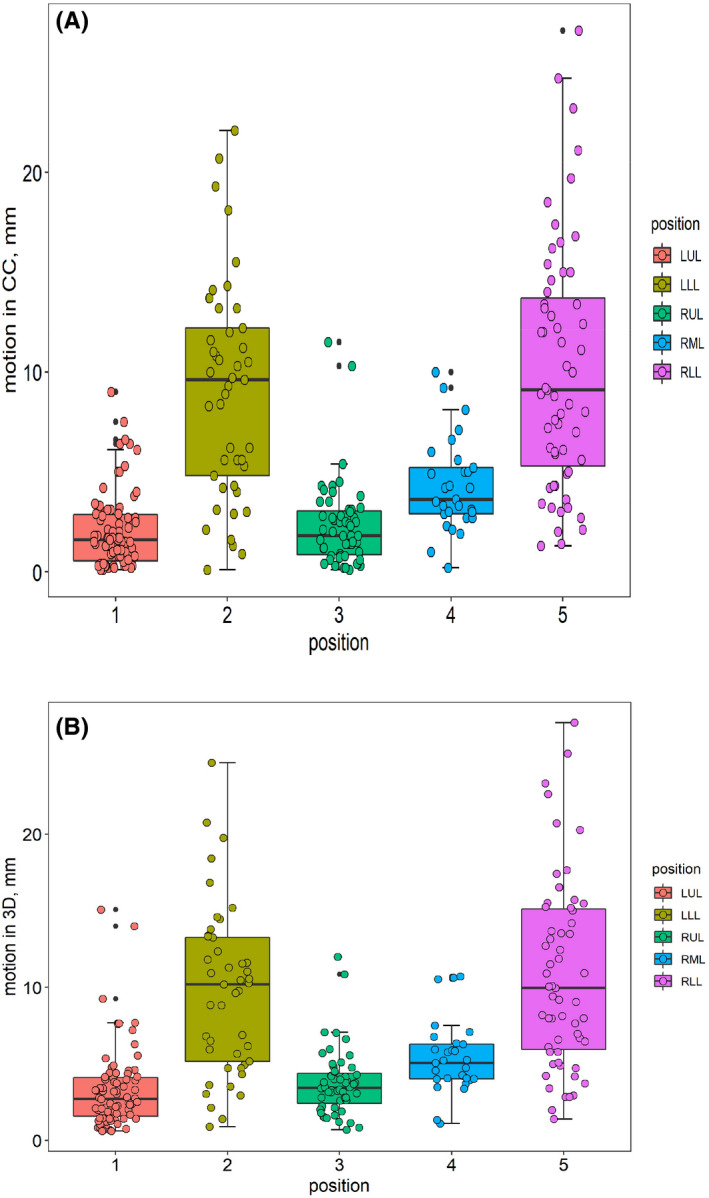
(A) The distribution of the motion in the CC direction grouped by the position of the tumor. (B) The distribution of the motion in the 3D direction grouped by the position of the tumor

Table 1 presents the baseline characteristics of study variables by the cancer pattern. Primary tumor subjects (n = 172) were more likely to be older stage I patients, with bigger tumor volume and larger tumor motion amplitudes in the LR, AP, CC, and 3D directions, and with more incidence of hypertension and cardiopathy than subjects with metastatic tumors (n = 95). Supplement Figure S1A,B represent the distributions of the tumor motion in the LR and AP directions, respectively, grouped by the tumor position. Tumors in LLL, RML, and RLL were more active than in LUL in both the LR and AP directions. Figure 1A,B present the distributions of the tumor motion in the CC and 3D directions, respectively, grouped by the tumor position. Compared with tumors in LUL, tumors in LLL and RLL presented a conspicuous larger amplitude of motion in the CC and 3D directions.

**TABLE 1:** Baseline characteristics of participants by tumor origin

Variable	Primary tumor	Metastatic tumor	Total	*p* value
N	172	95	267	
Male, n (%)	97 (56.40)	58 (61.05)	155 (58.05)	0.543
Age, y	65.55 (10.85)	59.66 (13.47)	63.45 (12.15)	<0.001
Smoker, n (%)	73 (42.44)	31 (32.63)	104 (38.95)	0.149
Stage, n (%)				
I	101 (58.72)	0 (0.00)	101 (37.83)	
II	7 (4.07)	0 (0.00)	7 (2.62)	
III	23 (13.37)	5 (5.26)	28 (10.49)	
IV	41 (23.84)	90 (94.74)	131 (49.06)	<0.001
Pulmonary surgery, n (%)	15 (8.72)	31 (32.63)	46 (17.23)	<0.001
Abutment, n (%)				
Solitary pulmonary	125 (72.67)	72 (75.79)	197 (73.78)	
Adhesion to parietal pleura	33 (19.19)	10 (10.53)	43 (16.11)	
Adhesion to mediastinum	6 (3.49)	6 (6.32)	12 (4.49)	
Adhesion to heart	8 (4.65)	7 (7.37)	15 (5.62)	0.185
LR, mm	1.30 [0.90, 2.10]	1.10 [0.60, 1.75]	1.20 [0.80, 2.10]	0.019
AP, mm	2.10 [1.20, 3.00]	1.60 [1.00, 2.15]	1.90 [1.10, 2.80]	0.008
CC, mm	3.45 [1.50, 8.53]	3.10 [1.45, 6.70]	3.20 [1.50, 7.95]	0.370
3D, mm	4.72 [3.01, 9.83]	4.01 [2.47, 7.42]	4.43 [2.81, 8.83]	0.131
GTV‐EE, mm^3^	5.25 [2.88, 13.05]	2.70 [0.98, 5.15]	4.20 [1.85, 10.30]	<0.001
Lobes, n (%)				
LUL	51 (29.65)	28 (29.47)	79 (29.59)	
LLL	32 (18.61)	13 (13.68)	45 (16.85)	
RUL	43 (25.00)	12 (12.63)	55 (20.60)	
RML	13 (7.56)	16 (16.84)	29 (10.86)	
RLL	33 (19.19)	26 (27.37)	59 (22.10)	0.016
Segments, n (%)				
S1	29 (16.86)	9 (9.47)	38 (14.23)	
S2	24 (13.95)	13 (13.68)	37 (13.86)	
S3	32 (18.61)	11 (11.58)	43 (16.11)	
S4	18 (10.47)	17 (17.90)	35 (13.11)	
S5	2 (1.16)	6 (6.32)	8 (3.00)	
S6	17 (9.88)	10 (10.53)	27 (10.11)	
S7	3 (1.74)	6 (6.32)	9 (3.37)	
S8	8 (4.65)	3 (3.16)	11 (4.12)	
S9	20 (11.63)	6 (6.32)	26 (9.74)	
S10	19 (11.05)	14 (14.74)	33 (12.36)	0.027
Zoning, n (%)				
Interior	37 (21.51)	17 (17.90)	54 (20.23)	
Intermediate	73 (42.44)	49 (51.58)	122 (45.69)	
Lateral	62 (36.05)	29 (30.53)	91 (34.08)	0.357
KPS	84.93 (5.96)	85.158 (6.50)	85.011 (6.15)	0.772
BMI, kg/m^2^	24.249 (3.07)	24.402 (3.02)	24.303 (3.05)	0.695
BSA, m^2^	1.745 (0.16)	1.79 (0.17)	1.761 (0.17)	0.033
Diabetes, n (%)	17 (9.88)	11 (11.58)	28 (10.49)	0.823
Hypertension, n (%)	49 (28.49)	12 (12.63)	61 (22.85)	0.005
Cardiopathy, n (%)	44 (25.58)	13 (13.68)	57 (21.35)	0.034
Pulmonary disease, n (%)	78 (45.35)	35 (36.84)	113 (42.32)	0.223

Abbreviations: AP, anterior–posterior direction; BMI, body mass index; BSA, body surface area; CC, cranial–caudal direction; LLL, left lower lobe; LR, left–right direction; RLL, right lower lobe; RML, right middle lobe; RUL, right upper lobe.

Table 2 represents the tumor motion of different lobes and segments. Tumors in LLL and RLL present the largest amplitude than other lobes, except in the LR direction. For different segments, the number of subjects who showed tumor motion was 38, 37, 43, 35, 8, 27, 9, 11, 26, and 33 for segment 1 to segment 10, respectively. The results of the analysis of variance show that a significant difference exists in tumor motion in all directions. The tumor in segment 7 was more active than others in the AP, CC, and 3D directions with the median [IQR] found to be 3.30 [2.80, 5.40], 14.60 [11.60, 15.50], and 15.47 [11.81, 16.84], respectively. Segment 5 in the LR direction was found to have the highest risk with the tumor motion median [IQR] found to be 1.85 [1.25, 2.93].

**TABLE 2:** Tumor motion of participants by tumor position (mm)

Variable	LR	AP	CC	3D
**Lobes, n (%)**				
LUL (n = 79)	1.10 [0.50, 1.60]	1.40 [1.00, 2.30]	1.60 [0.55, 2.85]	2.71 [1.58, 4.11]
LLL (n = 45)	1.20 [0.80, 2.10]	2.00 [1.50, 2.90]	9.60 [4.80, 12.20]	10.18 [5.17, 13.24]
RUL (n = 55)	1.30 [0.85, 1.85]	2.10 [0.90, 2.70]	1.80 [0.85, 3.05]	3.42 [2.42, 4.38]
RML (n = 29)	1.40 [0.90, 2.70]	1.80 [1.40, 2.50]	3.60 [2.90, 5.20]	5.06 [4.01, 6.28]
RLL (n = 59)	1.30 [1.00, 2.20]	2.30 [1.40, 2.95]	9.10 [5.30, 13.70]	9.95 [5.96, 15.10]
*p* value	0.085	0.013	<0.001	<0.001
**Segments, n (%)**				
S1 (n = 38)	0.90 [0.50, 1.38]	1.15 [0.83, 2.38]	1.30 [0.40, 2.08]	2.57 [1.35, 3.63]
S2 (n = 37)	1.00 [0.70, 1.80]	1.40 [1.00, 2.30]	1.70 [1.00, 2.70]	2.63 [1.54, 4.28]
S3 (n = 43)	1.40 [1.00, 1.85]	1.50 [1.00, 2.25]	1.60 [0.80, 2.80]	3.30 [2.35, 4.07]
S4 (n = 35)	1.30 [0.75, 2.60]	2.10 [1.55, 3.00]	3.60 [2.80, 5.10]	4.74 [3.97, 6.55]
S5 (n = 8)	1.85 [1.25, 2.93]	1.55 [1.38, 1.98]	3.15 [2.05, 6.05]	4.63 [3.81, 7.05]
S6 (n = 27)	1.20 [0.80, 2.00]	2.20 [1.50, 2.90]	4.90 [2.40, 7.65]	5.66 [3.33, 7.97]
S7 (n = 9)	1.00 [0.80, 1.30]	3.30 [2.80, 5.40]	14.60 [11.60, 15.50]	15.47 [11.81, 16.84]
S8 (n = 11)	1.40 [1.05, 2.10]	2.50 [1.90, 3.00]	8.90 [5.15, 12.35]	9.41 [5.78, 12.82]
S9 (n = 26)	1.70 [0.93, 2.20]	2.45 [1.43, 2.98]	10.70 [6.45, 14.08]	11.22 [7.25, 14.78]
S10 (n = 33)	1.30 [1.00, 2.50]	1.70 [0.90, 2.20]	10.80 [7.60, 13.70]	11.29 [7.99, 14.58]
*p* value	0.021	<0.001	<0.001	<0.001

Abbreviations: AP, anterior–posterior direction; CC, cranial–caudal direction; LLL, left lower lobe; LR, left–right direction; RLL, right lower lobe; RML, right middle lobe; RUL, right upper lobe.

To explore the influence of factors on tumor motion in different directions, multiple linear regression models were established. Backward stepwise regression was used to select variables. Table S1 shows the important factors of the tumor motion in the LR direction. Patients with metastatic tumors showed a small amplitude of motion while BMI was found to be a risk factor. Table S2 shows the variables that influence the tumor motion in the AP direction. The metastatic tumor also had a protective effect compared with the primary tumor. The results of tumor motion in the CC direction are presented (Table 3). Metastatic tumor and surgery were found to be protective factors, while BSA was found to be a risk factor. Compared with segment 1, segments 4–10 showed a larger amplitude of motion with the standardized partial regression coefficients found to be 1.10, 1.10, 1.14, 2.29, 1.61, 1.62, and 1.89, respectively. Compared with other clinical variables, the segment was the most important factor for tumor motion in the CC direction and segment 7 had the largest effect among the segments. Table S3 shows the important variables that influence the tumor motion in the 3D direction. The results were similar to the motion in the CC direction.

**TABLE 3:** Influence of factors on the tumor motion in the CC direction

Variables	Standardized estimate	Standard error	*p* value
Metastatic tumor	−0.31	0.125	0.013
Surgery	−0.34	0.152	0.027
Cardiopathy	0.22	0.136	0.112
BSA	0.13	0.055	0.020
GTV‐EE	−0.09	0.055	0.093
Lobes			
LUL	Reference	—	—
LLL	0.38	0.407	0.350
RUL	0.28	0.164	0.086
RML	0.40	0.304	0.186
RLL	0.67	0.384	0.083
Segments			
S1	Reference	—	—
S2	0.36	0.209	0.083
S3	0.30	0.197	0.128
S4	1.10	0.290	<0.001
S5	1.10	0.433	0.011
S6	1.14	0.433	0.009
S7	2.29	0.514	<0.001
S8	1.61	0.461	<0.001
S9	1.62	0.406	<0.001
S10	1.89	0.441	<0.001

Abbreviations: BSA, body surface area; LLL, left lower lobe; RLL, right lower lobe; RML, right middle lobe; RUL, right upper lobe.

Almost all results showed that tumor origin was an important variable. Stratification analyses were used to explore the difference in the tumor motion in the CC direction in the different subgroups. Table 4 presents the difference between patients with metastatic tumor and patients with primary tumor. In patients with metastatic tumors, almost all variables did not have any effect, except for surgery, which was a protective factor, while BSA, tumor position, and tumor segment were significant risk factors found in patients primary with tumors. We found that the tumor segment was also the most important factor while segment 7 had the largest effect, with the standardized partial regression coefficient of 1.97, compared to the other segments. The results for the comparison of surgery subjects and non‐surgery subjects were found to be similar (Tables S4 and S5).

**TABLE 4:** Influence of factors on the tumor motion in the CC direction by tumor origin

Variables	Metastatic tumor		Primary tumor	
	Standardized estimate	*p* value	Standardized estimate	*p* value
Surgery	−0.49	0.022	−0.25	0.301
Cardiopathy	0.51	0.080	0.13	0.404
BSA	0.07	0.496	0.21	0.003
GTV‐EE	−0.08	0.304	−0.06	0.483
Lobes				
LUL	Reference	—	Reference	—
LLL	0.50	0.527	0.62	0.220
RUL	−0.26	0.443	0.50	0.009
RML	0.55	0.244	0.26	0.518
RLL	1.07	0.161	0.84	0.081
Segments				
S1	Reference	—	Reference	—
S2	−0.15	0.712	0.51	0.041
S3	0.66	0.128	0.09	0.698
S4	0.62	0.221	1.31	<0.001
S5	0.63	0.360	1.16	0.064
S6	0.19	0.826	1.19	0.024
S7	1.75	0.059	1.97	0.006
S8	0.87	0.377	1.52	0.005
S9	1.28	0.084	1.39	0.008
S10	1.15	0.183	1.81	0.001

Abbreviations: BSA, body surface area; LLL, left lower lobe; RLL, right lower lobe; RML, right middle lobe; RUL, right upper lobe.

## 4 DISCUSSION

In this study, we assessed the tumor motion in different directions by measuring the peak‐to‐peak displacement of the COM of the 10 phases on 4DCT. Furthermore, we systematically evaluated the influence of target‐related and clinical factors on the tumor motion of 267 tumors in 246 patients. The data showed that the mean tumor motion in the LR, AP, CC, and 3D directions were 1.5, 2.2, 5.3, and 6.4 mm, respectively. The motion in the LR and AP directions was in line with results reported in other studies,^17^, ^25^, ^26^ while that in the CC and 3D directions was smaller than that reported in previous studies. Population differences may have led to the difference. The tumor motion in different lobes showed a significant difference in each direction; however, there was no significant difference in the tumor motion between the left and right lungs for the upper or lower lobe.

We further assessed the tumor motion in different lung segments and found that the motion in the CC direction for Segment 7 (S7) was the largest. The median motion was found to be 14.6 mm. Moreover, the motion in the CC direction for S9 and S10 was more than 10 mm, while the motion for S1–S3 was less than 3 mm. Each lung segment constitutes the basic morphological and functional unit of the lung, which represents the location of the lung in the 3D direction. We hypothesized that the segment location of the tumor might be an important influencing factor for tumor motion. Our data proved the hypothesis, and the tumor segment location was identified as a significant predictive factor for the tumor motion in each direction. Previous studies have reported that the tumor CC location was significantly correlated with the tumor motion, and considered as the primary factor. According to this study, the tumor segment location might be more convenient for clinical application and might hold a greater potential for assessing the tumor motion compared to the CC location.

Controversy persists regarding the impact of the tumor size on the tumor motion in previous literature.^9^, ^17^, ^20^, ^25^, ^27^, ^28^ A study of 152 patients by Liu et al^17^ reported that the tumor size was significantly related to the tumor motion in the CC direction. The same result was also shown in previous studies by Sarudis et al.^20^ and Adamczyk et al.^27^ However, several studies have contradicted this conclusion, and no significant correlation was found between the tumor size and motion.^9^, ^25^, ^28^ We investigated the influence of tumor size on tumor motion in a large study population using multiple linear regression analysis. Our study showed that the tumor size was a dependent predictive factor for tumor motion in the 3D and AP directions (*p* = 0.023 and 0.049, respectively), while it was not a dependent factor in the CC and LR directions. Additionally, we found that the predictive power of the tumor size was very low, and the standardized estimates in the 3D and CC directions were both found to be −0.08, which illustrated the discrepancy in the impact of the tumor size on tumor motion shown in previous studies. Our finding was reliable because we used multivariate analysis and included 17 target and clinical factors.

Furthermore, we systematically evaluated the influence of clinical factors on the tumor motion in each direction and quantified the predictive power of different factors using the value of the standardized estimate. Our results suggested that tumor origin and history of intrathoracic surgery were important influencing factors that affect tumor motion. The metastatic tumor was regarded as an independent influencing factor in all directions and showed good predictive power with standardized estimates ranging from −0.26 to −0.34. A history of intrathoracic surgery was a significant negative factor for tumor motion in the CC and 3D directions (standardized estimate: −0.34 and −0.25, respectively).

We first found that BSA exhibited a significant positive correlation with the motion in the CC and 3D directions. BSA is associated with physiologic and metabolic processes such as blood volume, heart exchange, and the size of vital organs such as the heart and lung.^29^, ^30^ Therefore, the above factors might influence the tumor motion, especially the size of the heart. The result indicated that the tumor motion in the CC direction might be different for Asian and Euro‐American populations. This finding also indicated a trend for tumors that coexisted with cardiopathy (these patients might have a larger size of the heart^31^, ^32^) to move more in the 3D direction (standardized estimate = 0.17, *p* = 0.054). In the LR direction, BMI was considered as a significant factor for the tumor motion, which suggested that the margin in the LR direction should be focused on obese patients. Other clinical factors were not found to correlate with tumor motion (e.g., smoking, stage, pathological pattern, and coexisting pulmonary disease).

Subgroup analysis was performed based on the tumor origin and history of pulmonary surgery. Our data showed that the tumor motion for metastatic tumors tended to be smaller than that for primary tumors, but significant differences were only found in the LR and AP directions (*p* = 0.019 and 0.008, respectively). Further studies indicated that a history of pulmonary surgery significantly reduced the motion in the CC direction for metastatic tumors. A larger proportion of patients who had undergone pulmonary surgery may show reduced tumor motion (32.63% vs. 8.72%). Moreover, coexisting cardiopathy likely improved the motion. Neither the tumor segment nor lobe location was a significant factor for the CC motion for metastatic tumors, which was different for the primary tumors. Therefore, when predicting the motion of metastatic tumors, it might not be sufficient to merely consider the tumor location; additionally, clinical factors should be considered. To date, there are no reports regarding the motion for metastatic tumors, but Yu et al.^28^ have reported that locally advanced stage tumors are less mobile than early stage NSCLC, and the main reason for this observation is that advanced stage tumors tend to be anchored in more established vascular structures. The motion for metastatic tumors showed a similar trend compared with advanced stage tumors.

For patients who had undergone pulmonary surgery, the tumor motion was significantly smaller in the CC and 3D directions than for patients without pulmonary surgery (*p* = 0.024 and 0.015, respectively), which may be associated with pulmonary inflammation, tissue adhesion,^33^ and lung volume reduction after pulmonary surgery. The tumor motion for these patients is more unpredictable and is poorly associated with the tumor location and clinical factors. It is especially important to individually account for the tumor motion for patients who have undergone pulmonary surgery.

Notably, the tumor motion measured by simulated 4DCT may not represent the real tumor motion during treatment.^34^ Irregular respiration patterns can change the tumor motion magnitude.^35^ Moreover, the centroid of tumors was used to represent the tumor motion, but the periphery of tumors might not be according to the centroid due to shape change.^9^

## 5 CONCLUSION

Herein, we first found that the tumor segment location was a good predictive factor for the tumor motion in all directions, and it was more convenient and precise for use in the clinical setting. A larger tumor tends to have a smaller motion, but the power of tumor size for predicting motion was very low. Patients with metastatic tumors or those who have undergone pulmonary surgery showed a smaller and more unpredictable tumor motion, which was poorly associated with the tumor location. It is especially important to individually account for the tumor motion for these patients. We also first found BSA, BMI, and coexisting cardiopathy had a positive correlation with the tumor motion in a certain direction. Clinical factors combined with targeted‐related factors can be used to predict the tumor motion for an individual. The predictive information contributes to generating a reliable patient‐specific ITV.

## CONFLICTS OF INTEREST

There are no conflicts of interest to declare.

## AUTHORS’ CONTRIBUTIONS

FXL and JBL contributed to the study design, the delineation and writing the manuscript, and the patient enrollment. YLQ and TTZ participated the data statistics and analysis and writing the manuscript. ZC and XS contributed to the patient enrollment.TZ participated in the study design and data statistics and analysis. All authors read and approved the final manuscript.

## Supporting information

Supplementary Material

## Data Availability

The datasets used and/or analyzed during this study are available from the corresponding author upon reasonable request.
