# Supplementary material for: Evaluation of lung tumor motion in a large sample: Target‐related and clinical factors influencing tumor motion based on four‐dimensional CT
Source: Cancer Med. 2021 Sep 14;10(20):7126–35. doi: 10.1002/cam4.4255 (PMC8525155; doi:10.1002/cam4.4255)
Supplement: Supplementary file 1 — Supplementary Material [file CAM4-10-7126-s001.doc]

**Supplement tables and figures**

**Table S1.** Influence factors of the tumor motion in the LR direction

| Variables | Standardized estimate | Standard error | *P* value |
| --- | --- | --- | --- |
| Metastatic tumor | −0.28 | 0.122 | 0.002 |
| Hypertension | 0.15 | 0.090 | 0.130 |
| BMI | 0.13 | 0.042 | 0.002 |
| **Lobes** | | | |
| LUL | Reference | - | - |
| LLL | −0.67 | 0.308 | 0.030 |
| RUL | 0.23 | 0.123 | 0.068 |
| RML | 0.24 | 0.229 | 0.303 |
| RLL | −0.46 | 0.292 | 0.120 |
| **Segments** | | | |
| S1 | Reference | - | - |
| S2 | 0.12 | 0.157 | 0.460 |
| S3 | 0.44 | 0.149 | 0.004 |
| S4 | 0.40 | 0.218 | 0.066 |
| S5 | 0.56 | 0.328 | 0.091 |
| S6 | 0.95 | 0.329 | 0.004 |
| S7 | 1.17 | 0.390 | 0.003 |
| S8 | 1.10 | 0.347 | 0.002 |
| S9 | 1.08 | 0.308 | <0.001 |
| S10 | 1.18 | 0.333 | <0.001 |

Abbreviations: BMI, body mass index; LLL, left lower lobe; RUL, right upper lobe; RML, right middle lobe; RLL , right lower lobe

**Table S2.** Influence factors of the tumor motion in the AP direction

| Variables | Standardized estimate | Standard error | *P* value |
| --- | --- | --- | --- |
| Female | 0.15 | 0.112 | 0.155 |
| Metastatic tumor | −0.34 | 0.081 | <0.001 |
| BSA | 0.09 | 0.060 | 0.121 |
| BMI | 0.08 | 0.052 | 0.143 |
| GTV-EE | −0.08 | 0.039 | 0.046 |
| **Abutment** | | | |
| Solitary pulmonary | Reference | - | - |
| Adhesion to parietal pleura | −0.07 | 0.107 | 0.515 |
| Adhesion to mediastinum | 0.26 | 0.191 | 0.174 |
| Adhesion to heart | 0.41 | 0.173 | 0.017 |
| **Segments** | | | |
| S1 | Reference | - | - |
| S2 | 0.26 | 0.140 | 0.066 |
| S3 | 0.03 | 0.139 | 0.808 |
| S4 | 0.52 | 0.145 | <0.001 |
| S5 | 0.21 | 0.229 | 0.364 |
| S6 | 0.56 | 0.155 | <0.001 |
| S7 | 1.13 | 0.232 | <0.001 |
| S8 | 0.54 | 0.208 | 0.010 |
| S9 | 0.53 | 0.153 | <0.001 |
| S10 | 0.22 | 0.148 | 0.132 |

Abbreviations: BMI, body mass index; BSA, body surface area

**Table S3.** Influence factors of the tumor motion in the 3D direction

| Variables | Standardized estimate | Std. error | *P* value |
| --- | --- | --- | --- |
| Metastatic tumor | −0.26 | 0.083 | 0.002 |
| Surgery | −0.25 | 0.100 | 0.012 |
| Cardiopathy | 0.17 | 0.089 | 0.054 |
| BSA | 0.11 | 0.036 | 0.003 |
| GTV-EE | −0.08 | 0.036 | 0.023 |
| Lobes | | | |
| LUL | Reference | - | - |
| LLL | 0.19 | 0.268 | 0.486 |
| RUL | 0.27 | 0.108 | 0.013 |
| RML | 0.23 | 0.200 | 0.260 |
| RLL | 0.38 | 0.253 | 0.130 |
| **Segments** | | | |
| 1 | Reference | - | - |
| 2 | 0.09 | 0.137 | 0.492 |
| 3 | 0.24 | 0.130 | 0.061 |
| 4 | 0.69 | 0.191 | <0.001 |
| 5 | 0.70 | 0.285 | 0.015 |
| 6 | 0.72 | 0.286 | 0.012 |
| 7 | 1.69 | 0.339 | <0.001 |
| 8 | 1.07 | 0.304 | <0.001 |
| 9 | 1.15 | 0.267 | <0.001 |
| 10 | 1.30 | 0.290 | <0.001 |

Abbreviations: BSA, body surface area; LLL, left lower lobe; RUL, right upper lobe; RML, right middle lobe; RLL , right lower lobe

**Table S4.** Influence factors of the tumor motion in the CC direction in surgery patients

| Variables | Standardized estimate | Std. error | *P* value |
| --- | --- | --- | --- |
| Metastatic Tumor | −0.31 | 0.366 | 0.405 |
| Cardiopathy | 0.14 | 0.430 | 0.745 |
| BSA | −0.05 | 0.190 | 0.799 |
| GTV-EE | 0.71 | 1.111 | 0.531 |
| **Lobes** | | | |
| LUL | Reference | - | - |
| LLL | −2.07 | 1.709 | 0.236 |
| RUL | −0.03 | 0.556 | 0.960 |
| RML | 0.92 | 0.697 | 0.196 |
| RLL | −0.92 | 1.581 | 0.567 |
| **Segments** | | | |
| S1 | Reference | - | - |
| S2 | 0.61 | 0.704 | 0.390 |
| S3 | 1.28 | 0.620 | 0.047 |
| S4 | 0.60 | 0.709 | 0.407 |
| S5 | 0.86 | 1.122 | 0.448 |
| S6 | 2.88 | 1.718 | 0.104 |
| S7 | 4.52 | 1.907 | 0.025 |
| S8 | 2.89 | 1.223 | 0.025 |
| S9 | 3.35 | 1.769 | 0.069 |
| S10 | 4.35 | 1.729 | 0.018 |

Abbreviations: BSA, body surface area; LLL, left lower lobe; RUL, right upper lobe; RML, right middle lobe; RLL , right lower lobe

**Table S5.** Influence factors of the tumor motion in the CC direction in non-surgery patients

| Variables | Standardized estimate | Std. error | *P* value |
| --- | --- | --- | --- |
| Metastatic Tumor | −0.27 | 0.137 | 0.052 |
| Cardiopathy | 0.20 | 0.144 | 0.175 |
| BSA | 0.15 | 0.059 | 0.010 |
| GTV-EE | −0.10 | 0.054 | 0.075 |
| **Lobes** | | | |
| LUL | Reference | - | - |
| LLL | 0.73 | 0.437 | 0.096 |
| RUL | 0.29 | 0.174 | 0.094 |
| RML | 0.17 | 0.351 | 0.632 |
| RLL | 0.86 | 0.414 | 0.040 |
| **Segments** | | | |
| S1 | Reference | - | - |
| S2 | 0.33 | 0.217 | 0.130 |
| S3 | 0.13 | 0.208 | 0.530 |
| S4 | 1.36 | 0.329 | <0.001 |
| S5 | 1.20 | 0.481 | 0.013 |
| S6 | 0.91 | 0.468 | 0.054 |
| S7 | 1.89 | 0.553 | <0.001 |
| S8 | 1.32 | 0.528 | 0.013 |
| S9 | 1.42 | 0.432 | 0.001 |
| S10 | 1.55 | 0.476 | 0.001 |

Abbreviations: BSA, body surface area; LLL, left lower lobe; RUL, right upper lobe; RML, right middle lobe; RLL , right lower lobe


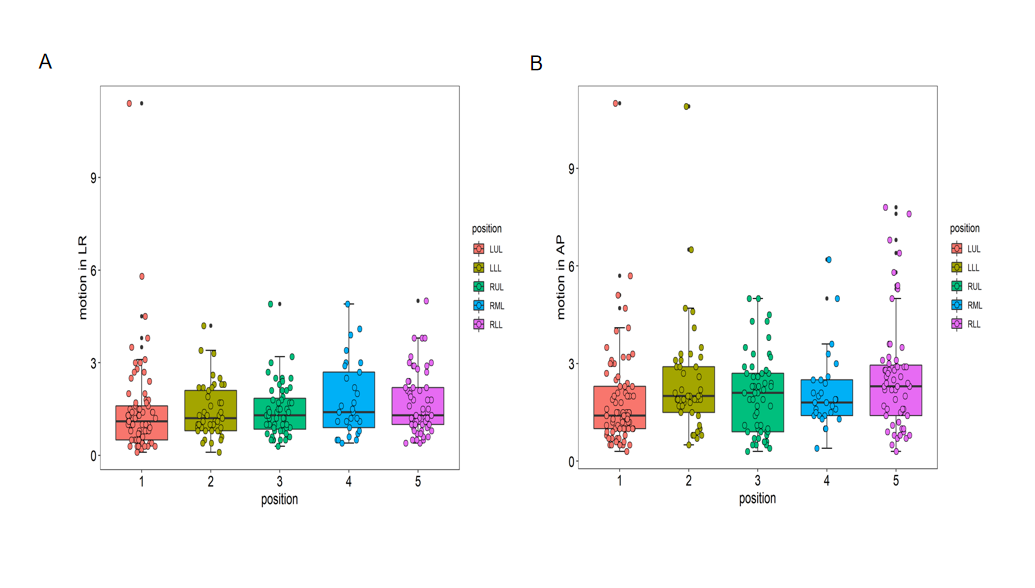


**Figure S1**. (A) The distribution of the tumor motion in the LR direction grouped by the tumor lobe location. (B) The distribution of the tumor motion in the AP direction grouped by the tumor lobe location.
